# Negotiating Identity and Belonging in a New Space: Opportunities and Experiences of African Youths in South Australia

**DOI:** 10.3390/ijerph17155484

**Published:** 2020-07-29

**Authors:** William Mude, Lillian Mwanri

**Affiliations:** 1 School of Health, Medical and Applied Sciences, Central Queensland University, Sydney, NSW 2000, Australia; 2 College of Medicine and Public Health, Flinders University, Adelaide, SA 5042, Australia; lillian.mwanri@flinders.edu.au

**Keywords:** identity discourse, integration process, resilience, resettlement challenges, CALD, African youths, Australia

## Abstract

This paper was part of a large study that aimed to explore determinants of increased suicides among African youths in South Australia. As part of this larger study, narratives from participants indicated that identity crisis could be a potential determinant of suicide. This paper reports on how African youths negotiate and form identity in Australia. A qualitative inquiry was undertaken with 31 African youths using a focus group and individual interviews. Data analysis was guided by a framework for qualitative research. These youths negotiated multiple identities, including those of race, gender, ethnicity and their origin. ‘Freedom and opportunity’, ‘family relationships’, ‘neither belonging here nor there’ and ‘the ability to cope against the paradox of resourcefulness in Australia’ appeared to be important themes in negotiating individual identities. An opportunity was used to acknowledge privileges available in Australia relative to Africa. However, the extent to which individuals acted on these opportunities varied, affecting a person’s sense of purpose, identity formation and belonging in Australia. The loss of social networks following migration, and cultural differences between African and Australian societies, shaped the experience of belonging and identity formation. These findings are crucial as they indicate the need for policies and practices that consider experiences of youths as they form their identity in Australia. Further studies with large numbers of participants are needed to explore these issues further among African youths in Australia.

## 1. Introduction

There is dearth of information about the mental health needs of Africans, especially young people in Australia, and most refugees do not present for mental health services. As part of the major study aimed to identify contributors of increased suicides among African youths in South Australia, themes including negotiation of identity strongly emerged from participants’ narratives. The formation of identity and the ways youths negotiated these in their new environment, in Australia, were further analysed and considered to be an important discovery, leading to the authors’ decision to unpack further and write this paper to contribute to the body of migrant and refugee health knowledge. Identity construction is an important process for migrant and refugee youths. It helps youths to construct realistic ambitions and reasonable ideals for themselves, develop a sense of free will and self-efficacy, and form a secure perception of self [1,2]. Additionally, planning for the future at this stage occurs within a social and cultural context that influences the ability of youths to engage in society successfully [2]. This process is likely to be particularly challenging for migrant and refugee youths given the additional complexities associated with negotiating identity in a new environment [3], often from a starting point of socioeconomic disadvantage [4]. Constructing and negotiating identity by migrant and refugee youths is concerned with social belonging and developing modalities of social relations in their new environment, as opposed to ethnic identity. Ethnic identity refers to how individuals relate themselves to a particular ethnic group through labelling, exploration, personal behaviours, and shared attitudes and beliefs [5]. There are complex links between constructing and negotiating identity and successful settlement outcomes. How a person negotiates their identity following immigration can have a significant impact on social and psychological adjustment in their new environment. A study suggests that forming a strong host-country identity is important in enhancing socio-cultural adjustment [6]. The same study reported that maintaining a strong country of origin identity is important for psychological adjustment. In some instances, identifying with heritage culture is protective against harmful behaviours [6] and improves academic performance [7] among youths. It is also worth noting that the effect can differ according to the nature of the heritage culture [8,9]. It appears that the ability to draw on the country of origin and the host country identity is vital to facilitate settlement in a new environment [10]. Evidence suggests that this acculturation strategy, referred to as ‘integration’ or ‘biculturalism’, is associated with higher self-esteem, lower rates of depression, and greater prosocial behaviours, especially among migrant youths [11]. However, ‘bicultural’ identity could present problems for a successful adaptation in a new environment. It is difficult for individuals to negotiate a bicultural identity when a sizeable cultural gap exists between the beliefs and practices of their country of origin and their host country. Additionally, there are issues that exist beyond individuals’ control, which shape their negotiating of identity [11]. For example, issues such as the cultural characteristics of interacting ethnic groups, socioeconomic status and social hierarchies, the availability of social and economic resources, and the socio-political aspects of the host country are vital dimensions that influence the negotiating of identity. The question of bicultural identity is particularly relevant for migrant and refugee youths in Australia. The first issue is that refugee youths often find it difficult to associate with a country of origin due to protracted stay in refugee camps. As a result, identity could become tied with their refugee status following such protracted situations [3]. Additionally, there is a difference in the racial construction of migrant and refugee youths and their peers in Australia, which shapes their cultural identity [12]. Moreover, the difference in cultural identity is formed by the effect of opposing individualist and collectivist forms of social organisation between migrant and Australian communities [13]. Although those immigrating to Australia may obtain national rights and access to services, many experience difficulties in connecting with Australian culture due to experiences of exclusion and cultural difference. Their experience is that of ‘guests’ in a foreign country [14]. Lastly, migrant and refugee youths focus on building identity and social networks with their new host community. This focus shapes the differences in acculturation between youths and the older generation in their community who are more likely to hold an identity associated with their place of origin [15].

It is well acknowledged that migrants and refugees generally undergo readjustment and adaptation on arrival to Australia, and initially experience decreased satisfaction when faced with the realities of life following their settlement. Evidence of phases of the refugee adjustment framework suggests that their ability to integrate with or become marginalised from Australian society depends on forces of social inclusion and exclusion, the existence of discrimination, and access to economic resources such as housing and meaningful employment [16]. It is of particular public health significance to understand how the social, cultural, political, and economic conditions of resettlement influence the negotiation of cultural identity among migrant and refugee youths.

Migrant and refugee youths are recognised as a vulnerable population group, especially to self-harm behaviours [17]. Additionally, refugee youths in particular are likely to have experienced multiple social stressors that make resettlement challenging. For example, the literature cites issues such as histories of trauma and abuse, loss of status and social networks, language barriers, unemployment, financial problems, and addiction to alcohol and substance use as challenges facing refugees following resettlement [4,17,18].

Although aspects of personal and group identity have been examined elsewhere [3,10,19,20,21], this study intended to explore this issue using an ecological perspective, [22] given the recognition of the socioeconomic and cultural challenges experienced by African migrant and refugee youths during resettlement in Australia, [4] and a desire to move beyond trauma-based understandings of refugee mental health and consider factors within the environment in which resettlement occurs, as echoed elsewhere [21,23,24].

This paper explores how African migrant and refugee youths in Australia negotiate cultural identity, given the limited understanding of the issue in the context of their local settlement. The aim of the paper is to better understand how cultural identity is negotiated and shaped among African migrant and refugee youths in South Australia. There are over 20,000 persons of African background in South Australia, and this number is increasing [25]. This understanding is important to inform health promotion practice and policy efforts to create appropriate environments that enable the healthy negotiation of cultural identity among migrant and refugee youths.

## 2. Methods

The current study employed a qualitative inquiry using both face-to-face interviews and focus group discussion. Qualitative research can reveal important accounts relating to social context, negotiating cultural identity and generating narratives that promote health-related behaviour discourse [26]. A focus group exploring the challenges of settlement and its impact on mental health was initially conducted, and the key issues from the results were used to inform further inquiry through semi-structured interviews.

### 2.1. Study Setting

The study took place in Adelaide, South Australia with 31 African migrant and refugee youths aged 18 to 25 years old. There were 17,784 migrant and refugee youths (aged 12–24 years old) residing within the Greater Adelaide region, which had a total youth population of 267,775 persons [27]. Of this number, over 30 percent of migrant and refugee youths resided within the northern region of Adelaide [27], an area of a relative socioeconomic disadvantage compared to the average for Greater Adelaide [28].

### 2.2. Recruitment and Data Collection

Research participants were opportunistically selected from a broader population of young people born in African countries and living in Adelaide, South Australia. Potential participants were approached by an African Youth Worker, employed by the African Communities Council of South Australia (ACCSA), who provided them with a written invitation to participate in the research. Before the commencement of focus group discussions and interviews, all study participants were provided with an information sheet outlining details of the research and its purpose. One focus group discussion with eight participants, and 23 interviews, were held at a central place where young people felt comfortable. The interview guide was informed by literature but also designed to allow flexibility to expand on points of interest and to explore issues that were considered important to individuals [29]. Interviews were conducted in English, as all participants spoke English. Ample time was set aside within each interview to establish rapport and to initiate and close the interviews in a sensitive manner. Interviews were confidential, and data was de-identified from the outset. Recordings of the focus group and interviews were transcribed verbatim professionally.

### 2.3. Data Analysis

Transcripts were analysed by two experienced qualitative researchers using the framework approach described by Ritchie and Spencer [30]. Framework analysis uses a systematic approach of data management to provide coherence and structure within the analysis process [30,31]. Passages of text representing repeated themes were identified and assigned headings according to the context and coded to several relevant categories to reduce the likelihood of missing key points. The data were then synthesised in sub-headings identified from the thematic analysis [30]. This approach is useful in enhancing the rigour, transparency and validity of the analytic process [32]. The analysis was both inductive, with categories emerging purely from the data and deductive, with categories derived from prior knowledge [33].

### 2.4. Ethical Considerations

All study participants were provided with an information sheet outlining details of the research and provided written consent to participate in the research. Some focus group members knew each other but not all. The issue of confidentiality was discussed with the focus group participants. Due to the sensitivity of the topic and the possibility of participants becoming distressed as a result of the focus group discussions and interviews, referral procedures were put in place prior to data collection to address the needs of distressed participants. After the data collection procedures were completed, all participants were provided with a list of professional counselling agencies where they could access support. Participants were offered a follow-up, private counselling session to discuss any issues that were raised by the interviews, with costs covered by the African Communities Council of South Australia (ACCSA). Each participant was reimbursed thirty dollars. Ethics approval was provided by the Flinders University Social and Behavioural Research Ethics Committee (SBREC project number 5480).

## 3. Results

As African migrants, many of the youths participating in this study experienced insecurity and displacement because of civil unrest in their countries of origin (Table 1). Social identity for these youths is beyond the physical construct that often shapes identity discourses. Identity for these participants was not just about their race (a social construct used here as a point of analysis), gender, ethnicity or country of origin. It is innate in nature and involved matters that are important for everyday life. There were multiple identities, and it relates to how they felt both in- and outside their physical body. The concept of ‘opportunity’ appeared to be important, regarded as a gift holding special meaning and privilege. As such, it came with the expectation that opportunity is used for reciprocating benefits to family in Africa and the Australian society more generally. However, the extent to which individuals acted on these opportunities in meaningful ways varied, affecting a person’s sense of purpose and belonging in Australia. Although there appeared a general feeling of being torn between two places among respondents, the loss of social networks following migration and cultural differences between African and Australian societies shaped the experience of belonging. The demographic details of the participants and the identified themes are presented with reference to salient quotations from respondents (Table 2).

### 3.1. Freedom and Opportunities

Respondents tended to recognise that the rights afforded to them upon settlement within Australia provided them with the opportunity to satisfy their personal needs and ambitions in a way that was not possible given their situation in Africa. This was expressed in terms of gratefulness, particularly considering the limited opportunities afforded to those still living in Africa. For example, there were views that showed a feeling of freedom from previous experiences of education.

In Australia, even though everywhere people have their own tough times and all that but I’m free here. No more running away from war. Free education. I have a lot of opportunities to do things that I want to do than when I was back home. (Respondent 23)

Then, I also have this opportunity where I can go to any university without being denied for my right to study … it’s a very important gift that God gave to us, so to me I think we are lucky to be here. (Respondent 17)

Through these views, not only did the participants perceive freedom as understood by the mainstream Australian community, but they also understood it in their own unique experiences. They linked freedom to their past experiences and integrated it to shape their self-construct in Australia. Freedom to study in Australia was particularly perceived as important for girls given experiences of girls’ education in Africa. The following quote shows how the freedom to education was perceived through a gendered lens.

It’s when you have freedom; you have the opportunity to study whatever you want, because that’s the hardest thing in Africa for a young girl, just to finish even year 11. To reach that stage it’s really hard where here you can study as many courses as you want, so it’s a privilege to be here, that’s what I always tell my friends. (Participant 12)

However, participants acknowledged that the availability of opportunities tended to be an insufficient condition for using those opportunities in an instrumentally meaningful and purposeful way. Rather, support external to the individual is required to use and benefit from these opportunities.

I think in Australia there are a lot of opportunities in education and all this kind of thing, but the downside of it is even if there’s education, if there’s no help to cope, you know, to cope with it then it becomes hard to use that opportunity. So, yes, there are opportunities, but we need help, something or someone to keep pushing us to get in there. (Respondent 14)

Some participants revealed pessimism because they felt their lives have not improved since coming to Australia despite the available opportunities in Australia. This view has profoundly shaped their self-outlook as expressed in the below quote.

When we were in Africa, life was really, really terrible and then when we come to Australia—me personally, I thought I was going to have a better life. I’ve been here for nine years now. I wake up every morning; the only thing I see shining is the sun, but my life’s not shining. (participant 14)

Additionally, many participants acknowledged that even if there are opportunities to study, they also experienced limited opportunities for employment, which negatively impacted their experience of living in Australia.

Then you try to get a job and then no-one offering you job, so you find yourself a bit depressed, I guess. Then if you find yourself a bit depressed, what do you do? Drink up, hang out with your friends, go out, make yourself feel good. (Respondent 21)

What I know so far, there are a lot of young people actually get frustrated from school and finding no jobs or getting an appropriate job, so things like that, and people who are actually going under the trauma of homelessness and into drugs, into other things which are negative actually to the young people (respondent 15)

### 3.2. Freedom and Family Relationships

Another common consensus among participants was around differences in culture from the older generation in relation to the rights and freedom of expression and how those disagreements came to shape the identity of some African youths in Australia. In some African cultures, youths are expected to follow the views of their parents and elders. Many youths in the study, however, perceived themselves as embodying Australian culture and were free to make an individual choice to do whatever they wish. Sometimes the ‘freedom’ in Australia put youths in the direct course of cultural conflict with parents and caused tensions within families. For example, the below quote captures this sentiment:

We are in Australia everyone is equal, and we know our rights and wrongs so that we argue with parents, like ‘this is wrong. This is what you’re supposed to say to me because you are wrong’ and then they don’t accept that. That’s how they take it to the community, because Africans consider that as being rude, you are not respecting your parents by talking back at them, but this is not what Australian culture says. Everyone is equal (Participant 12)

I’ve got pressure from my culture, like family at home, and then pressure from outside because I want to be—socially interact with others and they, my family, don’t want to accept that … I’m with my aunt here, and we have a lot of disagreement and stuff. Like you’re a girl, you don’t go out and meet friends except for close families, like a family that they know. Like I have friends from different places in Australia and stuff, but they don’t accept that they find it hard because they have different culture, like ‘no’. (Participant 10)

When participants were asked how the cultural conflicts within the family setting affected them, there was consensus around disengagements from their communities. Participants revealed that disengaged youths did not ‘fit’ in their communities or in the Australian culture. These missing pieces of identity from the country of origin and Australia made some participants define their own self-construct in ways that were comfortable and appropriate for themselves, for example, by going out to find a social group to identify with. Participants described the process of finding a sense of belonging in such a position as a “confused” situation. The following quote demonstrates this sentiment.

For me, I have to go to meet friend just to clear thing out … A lot of people, I think they isolate themselves from going out in the community or in a tribe with different people or getting involved in something … It always goes back to family, you know? Follow your culture with your family, listen to them, what they want you to do or just ignore them and do what you want to do to yourself. So, you’re between confused what-exactly how you’re going to help yourself to get out of that issue (Participant 10)

However, there remained tension between expectations for personal responsibility for action and the role of others in supporting this action. This reflected the recognition that personal freedom, representing the ability to define oneself and one’s own course of action, might result in very different consequences for an individual according to their personal resources and ability to seek out opportunities and support.

The good thing is that it’s you make your choices. At the end of the day, it’s up to you. Nobody makes the choices for you, but it’s up to you and if you’re willing to listen to anybody, take in their advice and sort of work out your life and stuff it’s up to you … You’re free, but all of that comes with consequences too. (Respondent 16)

### 3.3. Not Belonging and Being Torn between Two Places

Some respondents also noted conditions where opportunities could not be used in meaningful ways, making it difficult for individuals to find the means to enact their desired role within Australian society. The inability to fulfil personal ambitions resulted not only in a feeling of being trapped within Australian society, but also the experience of becoming marginalised from others because of barriers to engaging in meaningful education and employment.

Most youths are happy from outside, but inside they are not happy because they don’t have jobs. (Respondent 1)

Because you wake up every day and then, you know, you do the same thing, and you see the same people and the same things happening over and over again … You know you try to get—a job, trying to get to know people. Some of them tend to, you know, disengage from you and all that stuff. (Respondent 21)

The above quotations reveal the role employment opportunities and social relationships play in forming a meaningful self-concept of belonging. The inability to engage in employment or develop bonds with others led to the marginalisation and the experience of ‘not belonging’ by respondents. The experience of ‘not belonging’ can have a profound impact on an individual, as one of the participants demonstrated when narrating the experience of another youth in the community.

So, I know one of the persons who has committed suicide. Two weeks before he died there was kind of—he said ‘I want to go back to Africa’, you know? He told family ‘I want to go back. I don’t want to live here. This place is no good’. (Respondent 7)

Although it is not clear what the phrase ‘this place is no good’ means, other respondents made it clear that settling in Australia came with a feeling of being torn between two places, their country of origin and Australia. This is not to say that belonging is simply associated with connection to place, but also recognises that family and social ties may still exist within Africa and that there is a constant need to accommodate cultural beliefs from both African and Australian societies.

Yeah if you see—I have some people here you see—if you look at that you seem happy but inside you are not happy because I know—I have some cousins and my sister is here, but our mum is in Africa. (Respondent 1)

Let’s say if you want to marry now, you want a woman, you’ve got to have at least forty to sixty grands to marry the woman. You have to give them to the girl’s family and if you don’t have those you’re not going to get any girl. So that’s our—you know, it’s just our culture sometimes, it’s just different. Yeah, difficult. (Respondent 9)

Previously, it was mentioned that the inability to make use of educational and employment opportunities resulted in the experience of being trapped. Likewise, the experience of being trapped tended to emphasise the subjective feeling of not belonging, related to the perceived difference between African and Australian societies and a loss of family and social support networks.

Am I happy? I can say I am but like in the sense that I’ve got everything around me but in terms of that I’m not really—like I’m not really happy. Like back at home I was happy. Like we didn’t have food and all this stuff, but I still was happy. It’s just like maybe you can put it this way, here like you’ve been just surrounded by a fence around you, and you just want to escape, you know what I mean? (Respondent 21)

### 3.4. Using Available Resources

The study findings demonstrate that African youths in Australia draw on available resources when negotiating identities in Australia. For example, finding other youths from another community for social networks and a sense of belonging. They also draw on available services for youths in the community to maximise their access to resources that support their needs. Lastly, engaging in sport is another key activity through which African refugee youths negotiate their identities in Australia. Some participants who did not have their ‘ethnic’ communities in Australia revealed that they negotiated their identity through their friends in order to find a community for social activities and belonging. The following quote demonstrates this sentiment.

I don’t have my community here, like tribe; you know how Sudan, they have different tribes. So for me, I don’t have that community here so I get involved in other people’s communities, like other tribes. If they have program and stuff, I go there, just with my friends. (Participant 10)

The respondents in the current study also had a belief that there is a moral impetus not only to make use of available opportunities but also to actively seek support from others in obtaining and making effective use of those opportunities.

If you are youth here in Australia, you are a very young person, you get so many supports and so many help, and you should be seeking to get that help. There are a lot of sports activities. If you’re good at it Australian people will take you, you know. For example, if you want to make music and you’re really good at it, you can get a scholarship. If you’re good at basketball as African youth, you can get a scholarship. (Respondent 20)

Additionally, drawing on individual strengths of resilience and determination were important for the participants in this study. They acknowledged there were challenges of settling in Australia. In order to achieve their goals and contribute to the Australian community, however, participants emphasised the need to remain positive and focussed. The following two quotes demonstrate these views.

I think to be a young African youth in Australia—well, for me it’s a good gift, and it’s a great gift because no matter what happened to you if you’re still following your dream then I think it will be a good gift. (Respondent 17)

If you’re still alive, you are rich because one day you might become rich but if you’re dead, even though you are rich nobody will call you as rich because you are a dead person. That keeps other kids to push their lives, because they know one day, they will be rich. (Participant 11)

## 4. Discussion

This qualitative study attempted to further an understanding about how African youth negotiated their identity within urban settings of South Australia. Even though it is well acknowledged that the psychological construction of identity occurs throughout adolescence [1], youths, especially those with cultural backgrounds such as in the current study, require additional negotiations to incorporate their cultural, racial, and ethnic identity [3,5] within their new society. This is in recognition that, to migrants, migration to a new setting brings with it a range of novel challenges and stressors [34,35] compared to the general community in the host nation population. For youths settling into a new host culture, this also affects the social construct of their identity.

The findings of the current study revealed that African youths valued opportunities for self-determination, in particular the availability of formal education, freedom of speech and individual action, and freedom from oppression and war. The ability to realise personal ambitions was of significant importance for these youth. This is of particular relevance given that personal accomplishments and achievements have been found to predict personal and ethnic self-esteem among Australian immigrant youth, with personal self-esteem being the single major predictor of immigrant psychological health [19]. The presence of, and effective use of, opportunities enabled youth to realise ambitions and in doing so, promoted a sense of fulfilment and belonging. However, the ability to obtain and make use of opportunities was made difficult in the context of social exclusion and economic hardships. This study revealed that increased individual freedom would lead to different consequences depending on the motivations and resources of individuals. Because of the effect of economic disadvantage and the loss of social networks on arrival to Australia, social exclusion and economic hardship may limit individuals’ capacity to make use of opportunities, which in turn could further limit the attempts towards social inclusion and economic security. Within Australia, similar obstacles to social integration, including separation of family members, lack of access to housing and education support, and employment challenges have been observed amongst refugees and immigrants from the Horn of Africa [4]. Although discrimination and racism have been demonstrated to negatively affect an immigrant’s self-concept, self-esteem, and access to economic resources [3,16], this was not discussed by respondents within this study. However, this may have been a consequence of the action of more covert forms of discrimination due to the perceived cultural and racial difference between African migrant youths and their host society [14].

It is well acknowledged that upon resettlement, migrants, especially those with a refugee background, tend to pass through many phases of readjustment leading to outcomes ranging from integration through to marginalisation, with the direction of this outcome dependent on the presence or absence of resources in a person’s surrounding environment. Evidence also suggests that social inclusion, freedom from discrimination, and access to economic resources are important in promoting mental health during this resettlement process [16]. With its focus on African migrant and refugee youths, this study recognises the importance of these resources in resettlement, but conceptualises them as resources that can be drawn upon to realise their goals and ambitions, meet basic needs, and address the demands imposed by resettlement. This resonates with Dermot Ryan and colleagues’ ‘resource-based model of migrant adaptation’, which extends previous conceptualisations of migrant adaptation to recognise the role played by social environments in creating, or otherwise alleviating, stressful conditions impacting on resettlement. This is particularly relevant for those operating from a public health understanding as it takes the emphasis away from the role of individuals in managing demands and coping with stress and places it on the social conditions that create stressful demands and examines how social policies shape such conditions. Here, resources are the focus of our attention, including personal resources such as problem-solving and social skills, material resources such as housing and employment, social resources such as emotional and tangible support from others, and cultural resources that enable navigation of daily activities within a particular cultural environment. Importantly, it should be recognised that an individual’s resources are likely to be reduced, for various reasons, during each stage of the migration process, including resource losses endured prior to migration and during the migration process itself [24]. It is recognised that upon resettlement, migrants, especially those who are refugees, tend to arrive with histories of trauma and abuse, loss of status and social networks, language barriers, unemployment, financial problems, psychiatric disorders, and addiction to alcohol and drugs [4,17,18]. Thus, it becomes important to build the personal, material, social, and cultural resources of African youths upon arrival, attempting to replenish those lost during the process of migration. By building resources related to education and socioeconomic status, individuals will be more likely to successfully negotiate their own identity within a foreign country [36]. Our study has highlighted education, employment, and supportive social networks as resources important in the minds of African youth, enabling them to realise their needs and goals for life in Australia. It also suggests that a resource-based approach for enhancing adaptation of migrant youths provides a useful framework for public health action enabling youth to negotiate their own identity, alongside psychological, anthropological, and sociological understandings of identity formation [1]. A few limitations of this study are worth noting. One limitation is that the study was unable to elicit details regarding the nature of goals, needs, and demands experienced by these youth and the resources required to address them. It was also unable to identify the processes by which discrimination and racism impacted on the use of and access to resources, which is a key limitation given its potential to erode personal resources [3,16] and increase aversive demands [24]. Research shows that societal responses to refugees can act to preserve or undermine their human social resources and influence the experience of resettlement [37]. Furthermore, although we identified education as an important resource for African youths, we were not able to examine how this might relate to gaining meaningful employment and the impact of this on identity formation as a dynamic process [36]. This is an important consideration, as African migrants elsewhere have been observed to encounter significant barriers to finding employment related to English language comprehension and their ability to navigate job networks [38,39]. It was also less clear as to how histories of interrupted schooling and cultural and social language differences may have impacted on educational outcomes [40]. Further examination of these issues and the extent to which social policies can influence individual resources may prove useful in framing public health action to facilitate positive settlement outcomes for migrant and refugee youths.

## 5. Conclusions and Research Contribution to the Body of Knowldge

Attempting to understand the way in which the socio-cultural and economic conditions of resettlement influence the negotiation of identity among African refugee youth within urban South Australia, this study has highlighted the narratives between the meaningful use of life opportunities and the experience of fulfilment and belonging within Australia, with social and economic resources as necessary for individuals’ ability to make effective use of these opportunities. These findings call for consideration of the socio-cultural and political context surrounding resettlement and how this influences the negotiation of identity among migrant and refugee youths. They also resonated with a resource-based model of migrant adaptation, which shows promise in directing public health action, given its focus on social conditions that may promote or minimise psychological stress during the process of resettlement. Additionally, the recognition of African youths as a vulnerable group when it comes to negotiating identity is critical in understanding suicide in order to develop appropriate health policies and design effective interventions to prevent suicide among these and similar populations in Australia and elsewhere in settings where African migrants have been resettled.

Although this study is limited in its ability to examine the precise nature of resources that may act as enablers or barriers to the realisation of personal ambitions and needs, it has provided direction for future research aimed at examining these issues and how they might be addressed through public health policy. The contributions of this paper to the body of knowledge are as follows:

- Recognition of the ways young people negotiate identity is important as these internal negotiations can be partly contributory to determinants of suicide among young migrants in Australia.
- Nurturing support for African youths is necessary because when they develop their identity and adapt to changes in the new environment, this support can foster successful resettlement.
- Developing culturally sensitive social support services for African youths is necessary for a successful integration.

## Figures and Tables

**Table 1:** Characteristics of focus group youth participants.

No	Gender	Age at Data Collection	Country of Origin	Year Arrived in Adelaide
1	M	25	South Sudan	2000
2	F	20	Liberia	2005
3	F	21	Somalia	2001
4	F	23	Burundi	2005
5	F	20	Liberia	2005
6	F	23	Ghana	2004
7	F	24	Ethiopia	2005

**Table 2:** Characteristics of face-to-face interview participants.

Participants	Gender	Age at Data Collection	Country of Origin	Year Arrived in Adelaide
1	M	18	DRC	2010
2	M	20	South Sudan	2008
3	F	21	Liberia	2010
4	M	23	South Sudan	2009
5	M	20	South Sudan	2011
6	M	23	Ethiopia	2003
7	F	24	Liberia	2004
8	M	20	Burundi	2007
9	M	23	South Sudan	2007
10	M	25	South Sudan	2007
11	M	21	Liberia	2008
12	M	22	DRC	2005
13	F	25	South Sudan	2003
14	F	23	South Sudan	2005
15	M	20	South Sudan	2005
16	F	25	South Sudan	2008
17	F	21	Liberia	2008
18	M	25	South Sudan	2006
19	M	25	South Sudan	2003
20	M	20	South Sudan	2001
21	M	25	South Sudan	2006
22	F	18	Liberia	2005
23	F	23	South Sudan	2003
